# Potential mechanisms linking psychological stress to bone health

**DOI:** 10.7150/ijms.50680

**Published:** 2021-01-01

**Authors:** Jia-Sheng Ng, Kok-Yong Chin

**Affiliations:** Department of Pharmacology, Faculty of Medicine, Universiti Kebangsaan Malaysia, Cheras 56000, Malaysia.

**Keywords:** cortisol, hormones, mental stress, osteopenia, osteoporosis

## Abstract

Chronic psychological stress affects many body systems, including the skeleton, through various mechanisms. This review aims to provide an overview of the factors mediating the relationship between psychological stress and bone health. These factors can be divided into physiological and behavioural changes induced by psychological stress. The physiological factors involve endocrinological changes, such as increased glucocorticoids, prolactin, leptin and parathyroid hormone levels and reduced gonadal hormones. Low-grade inflammation and hyperactivation of the sympathetic nervous system during psychological stress are also physiological changes detrimental to bone health. The behavioural changes during mental stress, such as altered dietary pattern, cigarette smoking, alcoholism and physical inactivity, also threaten the skeletal system. Psychological stress may be partly responsible for epigenetic regulation of skeletal development. It may also mediate the relationship between socioeconomic status and bone health. However, more direct evidence is required to prove these hypotheses. In conclusion, chronic psychological stress should be recognised as a risk factor of osteoporosis and stress-coping methods should be incorporated as part of the comprehensive osteoporosis-preventing strategy.

## Introduction

Osteoporosis is a skeletal degenerative disease characterised by deterioration of bone microarchitecture and mass, which subsequently leads to fractures. Worldwide, 8.9 million osteoporotic fractures occur annually, which translate to a fracture every 3 seconds 1. Approximately 20 million Europeans aged over 50 years old suffered from osteoporosis, and 2.7 million experienced an osteoporotic fracture in 2017. This number is projected to increase by 23% in 2030. Financially, osteoporotic fractures incur substantial healthcare costs, amounting to 37.5 million euros in 2017 and is estimated to increase by 27% in 2030 2. The increasing prevalence of osteoporotic fractures calls for the need to identify high-risk individuals so that early intervention could be implemented.

Psychological stress occurs when an individual perceives an environment demand exceeding his/her ability to adjust. It can be measured as self-perceived stress and the effects of negative events. Chronic psychological stress is damaging to health because it produces long-term physiological, emotional, and behavioural changes that alter disease susceptibility 3. Chronic psychological stress is an emerging public health issue. In the United States, around $150 billion of revenue is lost annually due to stress-related behaviours, such as low productivity, absenteeism, poor decision making, stress-induced mental illness, and substance abuse 4. Besides, chronic psychological stress is a risk factor for various health conditions, such as heart diseases, asthma, obesity and diabetes 3. This review will focus on the effects of chronic psychological stress on bone health.

Affective disorders are the sequela of chronic psychological stress. Many studies have reported the association between affective disorders and bone health. Schweiger et al. first discovered the relationship between depression and osteoporosis, suggesting that affective disorders are associated with bone loss 5. Since then, the association between affective disorders and osteoporosis continues to be examined. Two meta-analyses have demonstrated that depression is associated with decreased bone mineral density (BMD) and increased fracture risk 6, 7. Catalano et al. reported that postmenopausal women with higher anxiety scores have lower BMD T-score 8. Populations with post-traumatic stress disorder also have a higher risk of developing osteoporosis in a longitudinal study 9. However, studies on the relationship between chronic psychological stress and osteoporosis remain limited 10. This gap is probably due to the assumption that these affective disorders are an extension of excessive psychological stress. However, individuals without affective disorders also experience psychological stress. Erez et al. found that even though the strength of the association between perceived stress and BMD is lower compared to depression in postmenopausal women, psychological stress still affects their bone health significantly 11.

Several studies have demonstrated that psychological stress is associated with osteoporosis 12-16. Fink et al. demonstrated that the number of stressful life events correlated positively with the risk of concurrent accelerated hip bone loss in older men 15. Besides, osteoporosis was found to be prevalent among UK veterans who participated in the Gulf War, probably due to chronic stress, including behavioural changes due to psychological stress during or after the war 17. Pedersen et al. also reported a positive association between the risk of osteoporotic fracture and perceived stress 18. Hahn et al. reported that lower BMD at the lumbar spine, femoral neck, and total femur was associated with moderate to severe perceived stress in men, but not for premenopausal or postmenopausal women 13. Given the existing evidence, one would ask how psychological stress induces bone loss. This review attempts to answer this question by providing a brief overview of the possible mechanisms involved.

## Factors mediating the relationship between psychological stress and osteoporosis

Psychological stress impacts bone health adversely through two prominent mechanisms, i.e. physiological changes and the attainment of unhealthy behaviours. It is important to note that the mechanisms discussed in this review affect the skeletal system inter-dependently, and their interactions should not be neglected.

### Physiological factors

Bone loss occurs due to the imbalance in bone remodelling when the rate of osteoclast-mediated bone resorption exceeds osteoblast-mediated bone formation. The bone remodelling process is mediated by the receptor activator of nuclear factor kappa-Β (RANK)/RANK ligand (RANKL)/osteoprotegerin (OPG) axis. RANKL released by the osteoblast lineage cells binds to its receptor, RANK, on the osteoclast precursor cells. The RANK-RANKL binding then stimulates the differentiation of osteoclast precursor cells into preosteoclasts, which fuse to form mature, multinucleated osteoclasts. Osteoblasts also secrete OPG, a decoy receptor for RANKL, which inhibits RANK-RANKL interaction and thus reduces osteoclastogenesis. Therefore, the ratio of RANK and OPG is indicative of the bone resorption rate 19.

Psychological stress is associated with the dysregulation of the endocrine system. Hypersecretion of cortisol, a key feature in chronic psychological stress, occurs due to dysregulation of the hypothalamus-pituitary-adrenal (HPA) axis 20-22. Cortisol binds to the glucocorticoid receptor, which undergoes conformational changes and translocates rapidly to the nucleus to bind to glucocorticoid response element and stimulate transcription of certain genes 23. Cortisol upregulates RANKL 24 and downregulates OPG expressions in osteoblasts 24-27. In murine cell culture studies, cortisol dose-dependently impairs osteoclastogenesis and osteoblastogenesis, and increases osteoblast and osteocyte apoptosis in cortical bone 28. In contrast, glucocorticoids promote the survival of osteoclasts 29. Overall, the increased glucocorticoid level may lead to negative bone turnover and subsequently bone loss. This mechanism explains the report of Henneicke et al., whereby even chronic mild stress upregulates glucocorticoid signalling, which subsequently causes bone loss, in a site- and gender-specific manner 14.

Furthermore, studies have shown that sustained psychological stress may originate from a pronounced and enduring hyperactivation of the sympathetic nervous system 30. Osteoblasts and osteoclasts possess receptors for neuropeptides and noradrenaline 31, implying the direct role of the sympathetic nervous system in bone homeostasis. Yirmiiya et al. demonstrated that stress-induced bone loss was associated with an elevated noradrenaline level in bone. They also reported that propranolol, a β-adregenic antagonist, may ameliorate stress-triggered osteoporosis. These observations suggest that hyperactivation of the adrenergic system might mediate the relationship between stress and bone loss 32. The level of neuropeptide Y, another neurotransmitter of the sympathetic nervous system, is reported to increase during mental stress and is associated with resilience to stress 33. Overexpression of neuropeptide Y inhibits bone formation and enhances bone loss 34. The action of neuropeptide Y on bone is mediated by hypothalamic Y1 and osteoblastic Y2 receptors 35. Neuromedin U is another neuropeptide synthesised in the hypothalamus, pituitary and small intestine, and is associated positively with stress behaviour and stress-related hormones in preclinical studies 36. Neuromedin U knocked-out mice were found to have increased bone mass due to increased bone formation. Further studies showed that neuromedin acted on bone cells through central nervous system but not directly 37. These neuropeptides may work together during chronic stress to induce bone loss.

Inflammation is one of the important secondary risk factors for osteoporosis, as observed in various inflammatory conditions 38, 39. Chronic psychological stress, as observed in the caregivers of ill patients, is associated with increased pro-inflammatory markers (PICs) 40. In preclinical studies, excessive inflammation of the brain impairs memory and learning, and induces depressive symptoms 41-43. Although both acute and chronic psychological stress are associated with increased in PICs 44, 45, prolonged exposure to PICs as in chronic stress poses a greater risk to health, including bone health. In bone remodelling, PICs promote RANK expression on monocytes, increase RANKL but reduce the production of OPG by osteoblast lineage cells, thereby increasing osteoclast formation 19, 46-49. In particular, tumour necrosis alpha-α is a more potent osteoclastogenic factor than the other PICs since it can amplify RANKL signalling in osteoclasts 19, 46-48, thereby enhancing osteoclastogenesis and bone resorption. Thus, low-grade chronic inflammation induced by chronic psychological stress might induce bone loss through increased bone resorption.

Many hormones secreted by the pituitary have direct and indirect actions on bone health 50. Prolactin is a pleiotropic peptide hormone produced by the lactotrophs in the anterior pituitary 51. Its primary function is to initiate and maintain lactation, but it also plays a significant role in stress response. Prolactin can stimulate the synthesis of catecholamines and sensitise the HPA axis 52. A wide range of different stressors, including ether 53, restraint 54-56, foot shock 57, 58 and noise 59, has been shown to elevate circulating prolactin levels in preclinical models. Osteoblasts but not osteoclasts express prolactin receptors 60. Prolactin exposure upregulates secretion of PICs (e.g. tumour necrosis factor-α and interleukin (IL)-1) in the osteoblasts and increases RANKL/OPG ratio, thereby favouring osteoclast formation and bone resorption 61, 62. The bone formation markers reduce in pups exposed to high prolactin level 63. However, in another study using co-culture of synovial fibroblasts and osteoclast progenitors, prolactin suppressed the formation of mature osteoclasts by inhibiting the secretion of RANKL by synovial fibroblasts 64. Thus, the effects of prolactin on osteoclast differentiation might depend on the adjacent cell types. On the other hand, increased prolactin level is associated with reduced BMD in humans 65, 66. Therefore, further studies are required to delineate the associations between psychological stress, prolactin level and bone health.

Variation in the lipophilic sex hormones that could penetrate the blood-brain barrier is suggested to affect the stress response of the brain. Women between puberty and menopause generally show lower HPA and autonomic axis activation during psychological stress than men, but the difference diminishes after menopause 67. Oestrogen supplementation after menopause attenuates HPA response of women during stress test 68. Despite this, Study of Women's Health across the Nation did not observe a significant increase in perceived stress and depressive symptoms during the menopausal transition 69, 70. However, Seattle Midlife Women's Health Study observed a significant increase in women's overnight cortisol levels during the menopausal transition, and the cortisol correlated significantly with sex hormones 71. On the other hand, menopause in women and testosterone deficiency syndrome in men are known risk factors for osteoporosis 72, 73. Gonadal hormones are essential in the maintenance of bone mass 74. Both oestrogen and testosterone play distinct roles in bone homeostasis in both sexes, and their actions are site-specific. In both men and women, oestrogen maintains 80% of the cortical bone mass. In women, oestrogen is the primary regulatory hormone of bone homeostasis for cancellous bone, while in men, testosterone maintains the cancellous bone mass 74. Oestrogens suppress osteoblast apoptosis and improve osteoblast survival. They also inhibit osteoclastogenesis by reducing serum RANKL level and promote osteoblastic production of OPG. Oestrogens also induce the production of Wnt/B-catenin, which promotes osteoblast maturation and increases OPG production through increasing transforming growth factor-β 74. Meanwhile, some actions of testosterone on bone health are attributed to its conversion to oestrogens by the aromatase enzymes 73, 75. Chronic stress reduces the serum level of both testosterone and oestrogen 76, 77. Thus, a reduction in gonadal hormones is partially responsible for psychological stress-induced bone loss.

Leptin is a peptide hormone synthesised by adipocytes to regulate food intake via the hypothalamus. Psychological stress-induced elevation in cortisol level increases the leptin level by stimulating leptin resistance 78, 79, though this action may be sex-dependent 80. Studies suggested a complex role of leptin in regulating bone remodelling. Leptin and leptin receptor-deficient hypogonadal mice showed increased trabecular bone volume at the spine but reduced bone mass at the femur 81-83. Intracerebroventricular infusion of leptin in wildtype mice and ewes reduced trabecular bone volume 83, 84. Takeda et al. demonstrated that antiosteogenic action of leptin was inhibited by gold thioglucose, which distorts the structure of ventromedial hypothalamic nucleus, demonstrating the involvement of the central nervous system in the action of leptin 85. Thus, increased leptin level during chronic stress could reduce bone mass through the central nervous system. On the other hand, systemic infusion of leptin generally protects against bone loss in animal models of osteoporosis 86-88. This observation probably explains the bone protective effects of obesity apart from increased mechanical loading since leptin level increases with increased fat mass. Therefore, the net skeletal effects of leptin during chronic psychological stress depend on the balance between the direct effects of leptin and its indirect effects through the central nervous system.

Parathyroid hormone (PTH) is an essential regulator and minute-to-minute determinant of both extracellular and intracellular calcium homeostasis in the blood 89. Using a murine model of restraint stress, Terzioqlu et al. demonstrated that psychological stress increases plasma PTH level by upregulating the calcium-sensing receptor on the chief cells in the parathyroid glands 90. The actions of PTH in the body are bi-directional 91. While intermittent exposure to higher than average PTH concentration exerts anabolic effects on the bone, sustained exposure to high PTH concentration can upregulate RANKL expression and inhibit OPG expression by the osteoblasts 92, 93. Therefore, sustained chronic stress may enhance bone loss via enduring, increased PTH concentration.

**Figure 1:**
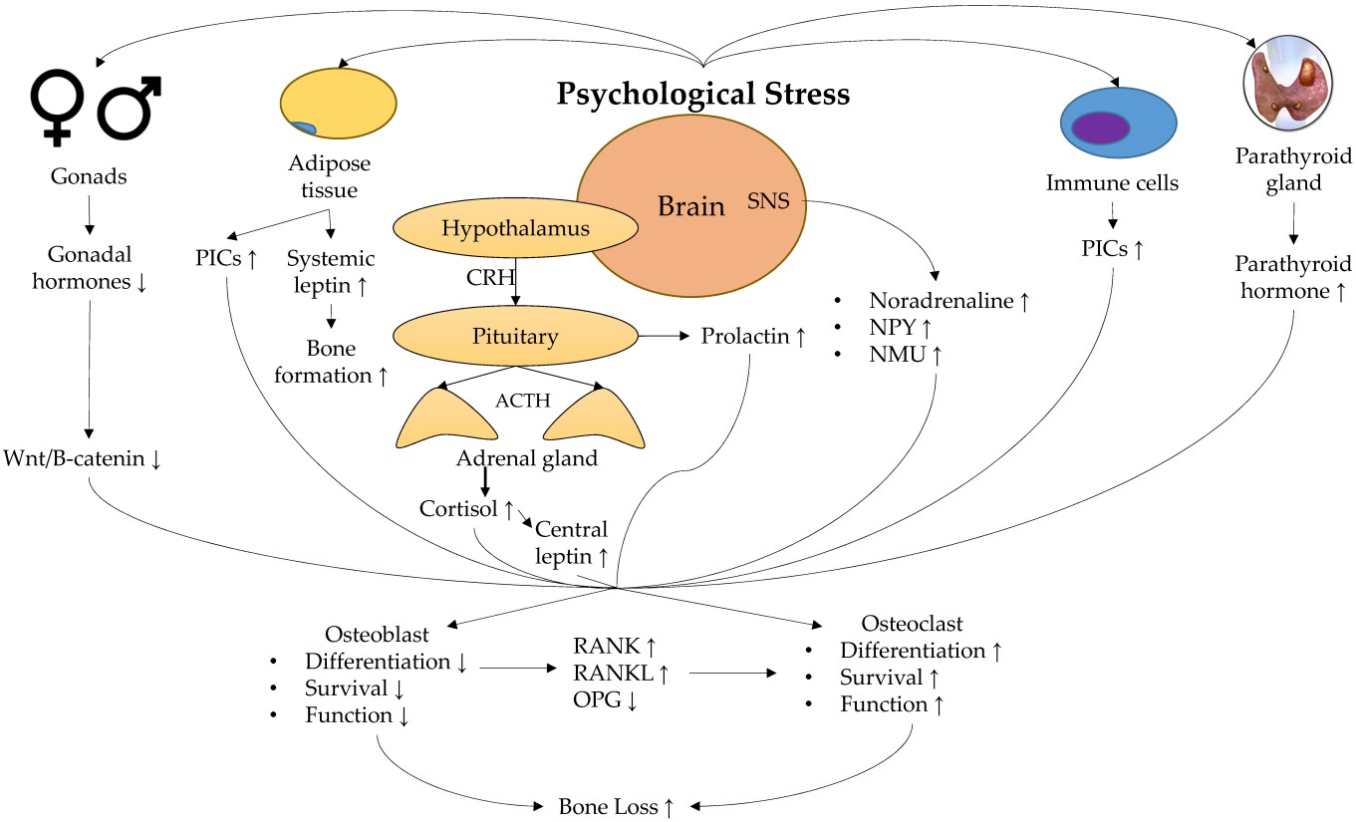
Physiological mechanisms linking stress and low bone mineral density. Abbreviation: ACTH, adrenocorticotropic hormone; CRH, corticotropin; NMU, neuromedin U; NPY, neuropeptide Y; OPG, osteoprotegerin; PICs, pro-inflammatory cytokines; RANK, receptor activator of nuclear factor kappa-B; RANKL, receptor activator of nuclear factor kappa-B ligand; SNS, sympathetic nervous system.

A summary of the physiological response towards psychological stress and its effect on bone is shown in **Figure 1.**

### Behavioural factors

In the basic stress model, stress can stimulate psychological (e.g. anxiety) and physical reaction (e.g. increased blood pressure) reactions, causing an individual to implement certain health behaviours to attenuate the reactions 94. Some of the common stress-coping behaviours include unhealthy eating habits 95, alcohol drinking 96 and cigarette smoking 97. In the Korea National Health and Nutrition Examination Survey (2007-2012), high calorie and alcohol intake, as well as cigarette smoking were associated with psychological stress and distress in both men and women 98.

Psychological stress has been proposed as a factor for smoking initiation and relapse, especially among women 99. Increasing stress due to income was associated with nicotine dependence in the lower-income group of Pennsylvania Adult Smoking Study 100. Despite some conflicting findings, smoking is generally agreed as a risk factor of osteoporosis 101. Nicotine, as the principal active ingredient in tobacco smoke 102, is reported to exert dual effects on osteoblast proliferation and function 103. Low-dose nicotine increases human mesenchymal stem cells proliferation and differentiation to osteoblasts, but high dose inhibits both processes 104. Nicotine also increases promote the secretion of PICs from osteoblast cultures 105, and in animal models 106. Of note, nicotine upregulates osteoblastic tumour necrosis alpha-α expression 105, which acts synergistically with RANKL to promote osteoclast formation 107. Non-toxic doses of nicotine could increase bone resorption activity of porcine osteoclasts in culture directly 108. In non-osteoblast suppressive doses, nicotine and cotinine (a metabolite of nicotine) inhibit catalase and glutathione reductase activity, leading to accumulation of ROS 109. Nicotine also suppresses sirtuin 3-mitochondrial superoxide dismutase 2 axis, leading to mitochondrial oxidative damage in osteoblasts 110. Apart from nicotine, tobacco smoke contains other chemicals. Exposure of mesenchymal stem/stromal cells to tobacco smoke suppresses osteogenic differentiation by increasing superoxide radicals and depleting intracellular glutathione 109.

Cigarette smoking is also associated with changes in hormone levels. Smoking status indicated by cotinine level has been associated negatively with thyroid-stimulating hormone level, but the relationship with thyroid hormones is not certain (triiodothyronine and thyroxine) 111, 112. TSH is associated with increased bone mass independent of triiodothyronine and thyroxine levels 113, so the decrease might impact bone health. However, a meta-analysis showed that smoking increased the risk of Graves' disease 114. Overt thyroid toxicosis is known to reduce bone mass through high remodelling 115. Smoking is also associated with higher cortisol in the morning and throughout the day in a cohort of elderly 116. The adverse effects of cortisol on bone have been elaborated earlier. In the Tromsø Study, higher serum PTH, lower vitamin D and calcium absorption were reported among smokers than the non-smokers of both sexes 117. Reduce calcium absorption will cause the body to mobilise the calcium reserve in the bone, resulting in bone loss. Lastly, smoking is associated with lower androgen levels in men 118, lower ovarian hormones in women with regular menses 119, and higher sex hormones in postmenopausal women 120. Therefore, the impact of smoking on bone mediated by sex hormones may depend on sex and menstrual status.

Perceived psychological stress has been associated with alternation of dietary habits. In a study involving Korean university students, higher perceived stress was associated with less frequent fruit and vegetable intake in both sexes and lower subjective judgement of healthy eating. Stress-related decrease eating was also apparent in these subjects 121. Similarly, higher perceived stress was associated with increased past-week soda, coffee, energy drink, salty snack, frozen food, and fast food consumption in Caucasian university students 122. In a study among Puerto Rican adults living in Boston, higher perceived stress predicted less fruit, vegetable and protein intake, but higher salty snacks 123. Prolonged practice of unhealthy dietary trend will have an impact on bone health. Higher fruit and vegetable intakes have been associated with increased BMD and reduced fracture risk 124, 125. This association could be mediated by the presence of vitamins and polyphenols in fruits and vegetables 126, as well as basic effects of the diet 127, which are beneficial to bone health. Vitamins and polyphenols act as antioxidants and anti-inflammatory agents, which promote osteoblasts survival and suppress osteoclast formation through mechanisms described in the earlier sections. High dietary salt intake is known to cause hypertension and increase urinary calcium excretion 128. A recent preclinical study also found that high salt intake caused induction of T-helper 17 cells and suppression of T-regulatory cells. The altered T-cell population led to increased PIC and reduced anti-inflammatory cytokine levels, in conjunction with the destruction of bone microstructure of mice on high salt intake 129.

On the other hand, psychological stress can lead to either reduced or overeating 121, 122. Long-term change of appetite could alter body weight. Multiple studies show that body weight is directly proportional to BMD 130, possibly via increasing mechanical loading of the body and synthesis of oestrogen by the adipose cells, which subsequently stimulate osteoblast activity 7. At the same time, adipose tissue is a source of PICs, which are detrimental to bone health 131. However, the association between chronic stress and body mass index remains unclear and confounded by age, gender, and race 132. In contrast, centralised obesity measured by waist-to-hip ratio shows a more consistent association with chronic stress 133. Centralised obesity is associated with activation of the HPA axis and dysregulation of the sympathetic nervous system, which results in bone loss 134. Thus, while increased BMI protects against bone loss, centralised obesity associated with psychological stress is detrimental to bone health. Underweight is consistently reported as a risk factor for osteoporosis due to reduced mechanical loading and malnutrition 135.

Alcoholism is prevalent among individuals with chronic stress due to the stress-relieving properties of alcohol. On the other hand, chronic alcohol use leads to altered stress-adaptive responses, triggering relapse and forms a vicious stress-alcohol use cycle 136. Heavy consumption of alcohol is detrimental to health, including that of the skeletal system 137. Alcohol affects bone health via many mechanisms, both direct and indirect 138, 139. Firstly, alcohol downregulates the expression of insulin-like growth factor 140, which enhances osteoblast maturation and proliferation 141. Secondly, chronic binge intake of alcohol upregulates the expression of PICs that stimulate bone resorption 142-144. Thirdly, the level of circulating sclerostin secreted by osteocytes was reported to be higher in alcoholics 145. Sclerostin inhibits Wnt signalling that regulates the osteoblast function, differentiation and survival, leading to decreased bone formation 145, 146. Fourthly, ethanol exposure upregulates the expression of NADPH oxidase enzymes that generate reactive oxygen species 147. These free radicals can enhance RANKL expression 148. Other adverse effects of alcoholism include decreased production of the gonadal hormones 149-151, decreased vitamin D level, which results in malabsorption of calcium, 139 and centralised obesity 152. All of these adverse effects can give rise to bone loss.

Psychological stress is associated with sedentary lifestyles 153, which in turns, are linked with low BMD 154, 155. Moreover, physical inactivity is associated with centralised obesity 156. This observation suggests that physical inactivity is one of the underlying mechanisms between psychological stress and low BMD. Physical activities have an important role in ameliorating chronic stress 157 and osteoporosis 158.

**Figure 2:**
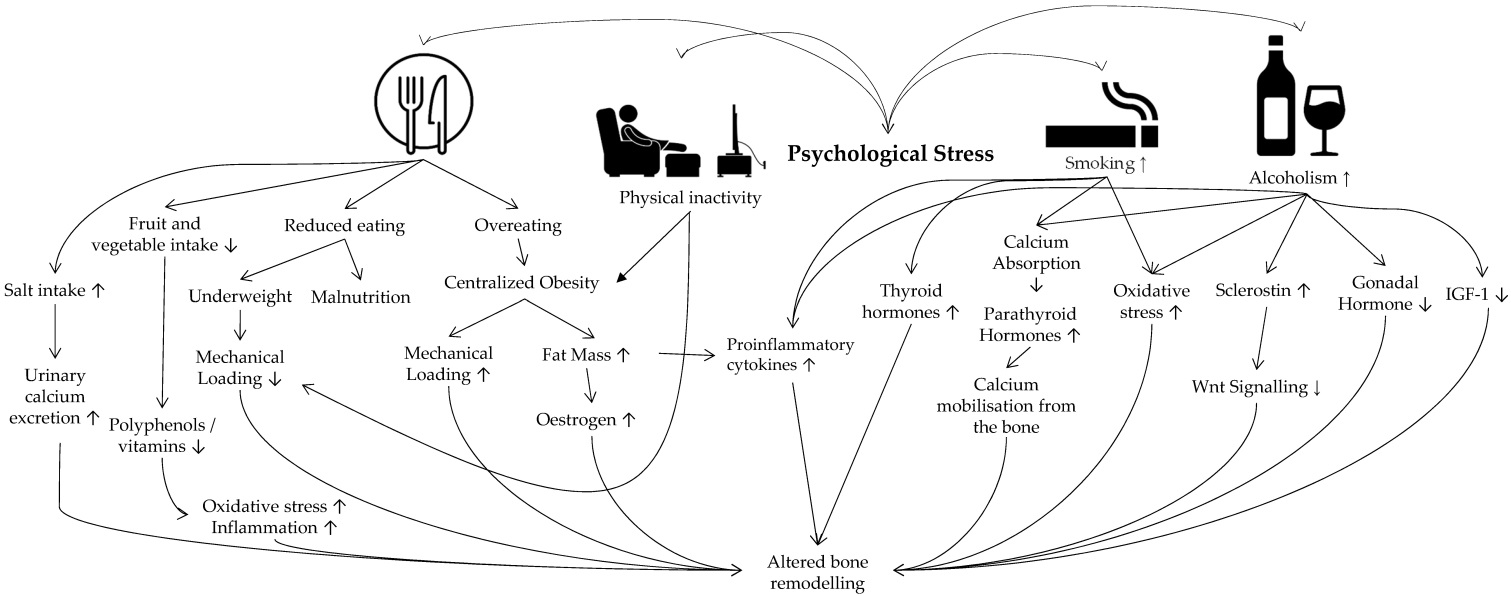
Behavioural factors linking psychological stress and osteoporosis. Abbreviation: IGF-1 insulin-like growth factors-1.

The overall effects of stress-induced behaviours on bone heath are presented in **Figure 2.**

## Epigenetic basis for the relationship between psychological stress and bone health

Epigenetic factors like DNA methylation also influence the development of skeletal diseases 159. Prenatal exposure of glucocorticoids induced by psychological stress could be responsible for epigenetic regulation of bone health. Prolonged glucocorticoid exposure in pregnant animals reduced mRNA expression of placental 11β-hydroxysteroid dehydrogenase-2 160, which regulates intrauterine exposure of the foetus to glucocorticoids. Restrain-induced stress in dams led to downregulation of gene expression of 11β-hydroxysteroid dehydrogenase-2 in the placenta, and altered gene expression of DNA methyltransferase of the brain in the foetus 161. Increased plasma corticosterone induced by diet restriction in dams also downregulated placental 11β-hydroxysteroid dehydrogenase-2 and transplacental exposure of the foetus to glucocorticoids. The alteration of HPA axis of the offspring is dependent on life stages, i.e. it decreases at weaning, changes marginally at young adulthood and is chronically hyperactivated at old age 162. These changes could alter skeletal development trajectory, but not many studies are available in the literature. In an animal study, mouse offspring born to dams subjected to restrain stress during pregnancy experienced increased corticosterone level, higher bone resorption, lower bone formation and significant deterioration of vertebral and femoral microstructures in adulthood 163. However, the epigenetic basis of these changes is not studied in detail. It should be noted that in animal models of stress, physical stressors (like restraint stress) is used, which may not represent the complexity of human psychological stress. Understanding the epigenetic regulation of chronic stress on the skeletal system is important, so that steps to protect bone health can be initiated in utero.

## Socioeconomic status and osteoporosis: is psychological stress a mediator?

The effects of psychological stress may provide a biological explanation of the relationship between socioeconomic status and bone health. A low economic status could lead to poor residential condition, community hazards, suboptimal access to medical support and malnutrition, which causes psychological stress and harms the general well-being of a person 164. Indices of socioeconomic status, such as household income 165, education level 72, living condition 166, have been associated with variation of BMD. A systematic review suggested a strong relationship between living with others and reduced risk of osteoporotic fracture, while evidence for income and education level is conflicting 167. Early-life exposure to income equality predicted life dissatisfaction and psychomotor symptoms among adolescents, especially among females 168. Whether this stress related to socioeconomic status is partially responsible for a higher uptake of behaviours detrimental to bone health among adolescents from lower socioeconomic, such as smoking and alcohol 169, 170, deserve further study. However, direct evidence proving the role of psychological stress as a mediator in the relationship between bone health and socioeconomic status is limited and should be validated in future studies.

## Conclusion

**Figure 3:**
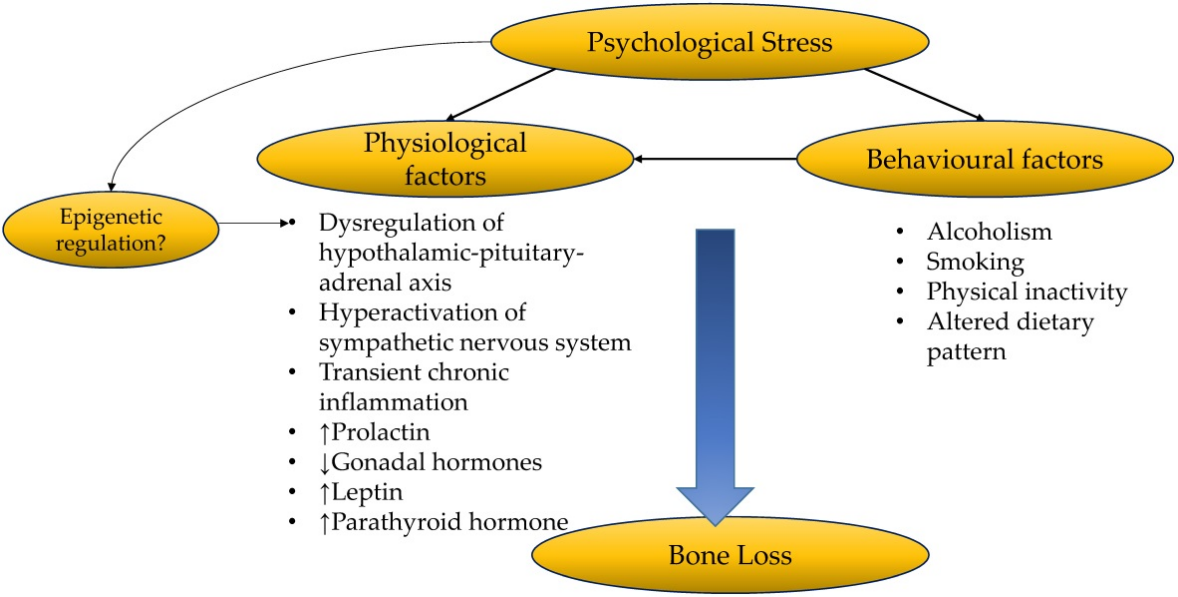
The relationship between psychological stress and bone health.

Psychological stress gives rise to complex physiological and behavioural changes which could affect bone health. These factors interact with each other to alter bone remodelling, resulting in a net bone loss (**Figure 3**). More comprehensive and well-planned epidemiological studies are needed to investigate the causal relationship between psychological stress and BMD or fracture. Future studies should take into consideration changes in biomarkers of inflammation, oxidative stress, hormones and neurotransmitters, to establish the relationship between psychological stress and bone health. The difference in the effects of acute/chronic stress, mild/moderate/chronic stress on the severity of bone loss should also be investigated. Lastly, stress management strategies should consider the multidimensional effects of health. Effective stress management should incorporate counselling and lifestyle interventions to prevent psychological stress to progress into affective disorders, and its related complications on other biological systems. For example, moderate exercise could have a positive impact in relieving psychological stress and improving bone health. A balanced and healthy diet will attenuate the enhancer of psychological stress such as neuroinflammation and oxidative stress, and promote optimal bone health.
